# Practice of pharmaceutical care by community pharmacists in response to self-medication request for a cough: a simulated client study

**DOI:** 10.1186/s12913-023-09642-x

**Published:** 2023-06-20

**Authors:** Pengyeow Loh, Jason W. Lee, Mahmathi Karuppannan, Siew Siang Chua

**Affiliations:** 1 School of Pharmacy, Faculty of Health & Medical Sciences, Taylor’s University, 1, Jalan Taylors, Subang Jaya, Selangor, 47500 Malaysia; 2 Department of Clinical Pharmacy, Faculty of Pharmacy, Universiti Teknologi MARA Selangor Branch, Puncak Alam Campus, Bandar Puncak Alam, Selangor, 42300 Malaysia

**Keywords:** Community pharmacist, Cough, Counselling, Pharmaceutical care, Simulated client

## Abstract

**Background:**

Community pharmacy practice worldwide has been shifting from product-focused to patient-oriented. However, due to the absence of separation between prescribing and dispensing in Malaysia, community pharmacists may have limited roles in the provision of pharmaceutical care to patients with chronic diseases. Therefore, the main functions of community pharmacists in Malaysia are related to self-medication requests for minor ailments and the supply of non-prescription medications. The objective of this study was to determine the practice of pharmaceutical care by community pharmacists within the Klang Valley, Malaysia in response to self-medication requests for a cough.

**Methods:**

This study utilised a simulated client method. A research assistant, acting as a simulated client, visited community pharmacies in the Klang Valley, Malaysia to consult the pharmacists on the treatment of a cough experienced by his father. Upon leaving the pharmacy premise, the simulated client entered the pharmacist’s responses in a data collection form which was structured based on pharmacy mnemonics for the response to symptoms, OBRA’90 on counselling elements, the five practice principles of pharmaceutical care by the American Pharmacists Association and literature review. Visits to the community pharmacies were conducted from September to October 2018.

**Results:**

The simulated client visited a total of 100 community pharmacies. None of these community pharmacists practised adequate patients’ data collection, with only a low proportion who practised all the components studied under medication information evaluation (13%), formulating a drug therapy plan (15%) and monitoring and modifying the plan (3%). Of the 100 community pharmacists, 98 recommended treatment but none of them provided all the counselling elements studied in implementing the drug therapy plan.

**Conclusion:**

The present study showed that community pharmacists within the Klang Valley, Malaysia were not providing adequate pharmaceutical care services to patients seeking self-medication for a cough. Such practice may compromise patient safety if inappropriate medicines or advice are given.

**Supplementary Information:**

The online version contains supplementary material available at 10.1186/s12913-023-09642-x.

## Background

Community pharmacy practice worldwide has shifted from product-focused to patient-centred, to achieve definite outcomes that improve a patient’s quality of life. This pharmaceutical care concept was first introduced by Hepler and Strand in 1990 [1]. The American Pharmacists Association (APhA) had developed five principles for the practice of pharmaceutical care. These are: patients’ data collection; medical information evaluation; formulating a drug therapy plan; implementing the plan; monitoring and modifying the plan [2].

In Malaysian government hospitals, the medical doctors prescribed and the pharmacists screened and dispensed the medications as well as identified and resolve pharmaceutical care issues through Medication Adherence Clinic (MTAC), working in collaboration with the medical doctors to monitor the patients’ clinical outcomes [3–5]. However, the absence of regulations on separation between prescribing and dispensing in Malaysia limits the community pharmacists’ opportunity to provide pharmaceutical care services as most patients will have their prescribed medications filled at the hospitals or private clinics instead of community pharmacies [6, 7]. Various local studies had also raised concerns about the under-utilization of community pharmacists in providing optimal care to the general public and contributing to the health care services of the country [6, 8, 9].

The Malaysian government’s national report on the use of medicines by consumers in 2015 showed that although medicines were widely used by the general public, 46.8% could not differentiate between the trade and generic name of their medicines, and 33.9% reported sharing their medications. In addition, 29.7% were not aware of the side effects, 18.6% did not fully understand the purpose of the medicines and 17.0% did not know about the proper storage of medicine. The study also highlighted that 70.8% of the consumers wanted additional medicine counselling from the pharmacists to understand and overcome the problem related to their medications [10].

Community pharmacists are the most easily accessible healthcare providers to the general public and hence, can play a major role in providing adequate information on the use of medicines [11, 12]. Provision of pharmaceutical care by community pharmacists is important especially when self-medication has become increasingly common, especially in urban areas such as the Klang Valley in Malaysia [13]. This may be attributed to changes in social-economic status, availability of health information and medicinal products [14].

Various studies on Malaysian community pharmacists have been conducted but mainly focussed on information and advice given through medication counselling [8, 15–17]. However, studies on the provision of pharmaceutical care by community pharmacists in Malaysia based on an international standard such as the five practice principles of pharmaceutical care by APhA, in response to the self-medication request are still lacking.

Therefore, the objective of the present study was to determine the practice of pharmaceutical care by Malaysian community pharmacists in response to a cough, for which patients often seek self-medication advice and treatment at community pharmacies. The findings of this study will enable healthcare stakeholders to better understand the practice of pharmaceutical care by community pharmacists. In addition, the study will bring awareness to community pharmacists on aspects of pharmaceutical care practice which are lacking and for them to better equip themselves to contribute optimally to the health care system of the country.

## Methods

### Study population

The target population was community pharmacists who were working on a full-time basis in community pharmacies in the Klang Valley, Malaysia. The Klang Valley is an urban agglomeration which includes Kuala Lumpur and its surrounding cities and towns, with an estimated population of 7.564 million [18] and the main business hub in Malaysia. It also has the highest number of community pharmacies [19]. Pharmacists who were working in the administration or wholesale department of a community pharmacy, or in a community pharmacy which is part of a hospital premise, were excluded. The present study utilised the simulated client method (SCM) where one simulated client (SC) visited all the selected community pharmacies to gather the information required.

### Simulated client’s scenario

A simulated case for this study was developed based on the literature [15, 20], mnemonics for response to symptoms [21] and with inputs from four independent experienced practising community pharmacists. The simulated scenario comprised of one SC who consulted the community pharmacists for the treatment of a dry cough experienced by the SC’s father. The father was a 60-year old taxi driver and a smoker. He had hypertension, hyperlipidaemia and diabetes mellitus, and was taking an angiotensin-converting enzyme inhibitor (ACEI), perindopril as the anti-hypertensive medication. This simulated scenario was created to determine if the pharmacists would ask appropriate questions to identify the possible causes of a cough which may appear to be a minor health problem, but may be due to other causes such as smoking or ACEI-induced [22]. A guide with standard answers to possible questions asked by the pharmacists was developed for the SC to follow (Additional file 1).

### Data collection form

The data collection form for the study was designed based on the five principles of pharmaceutical care practice by APhA [2]. The five practice principles of pharmaceutical care by the American Pharmacists Association are: (1) Patients’ data collection, (2) Medical information evaluation, (3) Formulating a drug therapy plan, (4) Implementing a drug therapy plan, and (5) Monitoring and modifying the plan [2]. These five practice principles were evaluated using 25 components in the data collection form (Additional file 2). Possible questions that the pharmacists might ask the client were structured based on mnemonics such as WWHAM (**W**ho is the patient; **W**hat are the symptoms; **H**ow long have the symptoms been present; **A**ction taken; **M**edication being taken), ASMETHOD (**A**ge/appearance; **S**elf or someone else; **M**edication; **E**xtra medicines; **T**ime persisting; **H**istory; **O**ther symptoms; **D**anger symptoms), ENCORE (**E**xplore; **N**o medication; **C**are; **O**bserve; **R**efer; **E**xplain) and SIT DOWN SIR (**S**ite or location of sign/symptom; **I**ntensity or severity; **T**ype or nature; **D**uration; **O**nset; **W**ith other symptoms; a**N**noyed or aggravated by; **S**pread or radiation; **I**ncidence or frequency; **R**elieved by) [21]. Counselling elements in the data collection form were developed based on the Omnibus Budget Reconciliation Act, 1990 (OBRA’ 90) from the United States of America, which requires pharmacists to provide medicine use review and counselling to the patients [23].

The content validity of the data collection form was reviewed and assessed by four practising community pharmacists with the assurance of confidentiality. In addition, another ten practising community pharmacists with 10 to 20 years of working experience, were consulted on which components must be fulfilled to determine the practice of each pharmaceutical care principle. The results were analysed and discussed and a consensus was reached by the research team which consisted of two experienced academicians who are registered pharmacists and an experienced community pharmacist (Additional file 2).

A pilot study was conducted on another 10 community pharmacies to test the feasibility of the study procedure and to ensure that the data collection form was able to capture all the information required. In addition, the pilot study allowed the SC to become familiar with the study procedure and the data collection form. The data collection form was modified slightly before being used in the main study. The results of the pilot study were excluded from the main study.

### Data collection procedure

There were 708 community pharmacies in the Klang Valley at the time of this study [24]. These community pharmacies were stratified based on their municipality and the number of community pharmacies selected from each municipality was proportional to the total number of community pharmacies in that municipality [24]. The community pharmacies were then selected at random based on random numbers generated by the Statistical Package for the Social Science (SPSS) software version 21.0 (IBM Corp, Armonk, New York). This study included only 100 community pharmacies based on previous SCM studies [15, 25]. However, a total of 150 community pharmacies were selected with an additional 50 to act as a buffer when a selected pharmacy did not have a pharmacist present during the SC’s visit or the pharmacy had ceased operation or did not meet the inclusion criteria.

The SC visited each community pharmacy and asked to see the pharmacist. If the pharmacist was present, the first question the SC asked was: “*Can I have something for cough?”.* The SC did not provide any other information unless he was asked by the pharmacist. If the pharmacist did not ask any further questions, the SC would probe the pharmacist with the following question: “*Is the medicine suitable for my father?”* This was to prompt the pharmacist to gather more information before providing any recommendation and to provide a basis for medication counselling. The SC would note the duration of consultation with the pharmacist by using the stopwatch function from his mobile phone while listening carefully to the pharmacist’s response, including questions asked and counselling points provided. At the end of the interaction with the pharmacist, the SC asked for the pharmacist’s name and name card to verify if the pharmacist was working full-time and also observed the pharmacist’s displayed name. In addition, the SC asked about the pharmacist’s duty schedule, in anticipation of future follow-up. Upon leaving the pharmacy, SC would immediately enter the information gathered from the encounter with the pharmacist in the data collection form. The SC visited the selected community pharmacies in the various municipalities until a total of 100 community pharmacies from those selected were covered.

### Data analysis

All data were entered into and analysed using SPSS version 21 (IBM Corp, Armonk, New York). Descriptive statistics were generated for all the variables, with mean, standard deviation (SD) or median and interquartile range (IQR) for numeric or scale data. Associations between characteristics of community pharmacists or pharmacies and the number of questions asked or counselling elements provided were first analysed using Univariate Poisson Regression under the Generalised Linear Model function. Variables with p value equal to or less than 0.25 were further analysed using Multivariate Poisson Regression [26]. A p value less than 0.05 was considered statistically significant.

## Results

The SC visited a total of 132 community pharmacies but only 100 community pharmacies were included in the present study. The remaining 32 community pharmacies were excluded due to various reasons: pharmacists were on leave or not available during the visits (24) and pharmacies closed or ceased operation (8). Characteristics of the 100 community pharmacists and pharmacies visited by the SC are shown in Table 1.

**Table 1:** Characteristics of the community pharmacists and pharmacies (N = 100)

Characteristics	Percentage
**Sex**	
Female	62
Male	38
**Location**	
Ampang Jaya	5
Selayang	7
Klang	8
Shah Alam	8
Kajang	9
Petaling Jaya	12
Subang Jaya	13
Kuala Lumpur	38
**Ownership**	
Individual non-pharmacist	2
Individual pharmacist	42
Group of pharmacists	33
Corporate bodies	23
**Number of branches in Malaysia**	
1	33
2 to 10	23
11 to 50	19
51 to 100	8
101 to 200	9
400 to 500	8

### Provision of pharmaceutical care services

The practice of the five principles of pharmaceutical care by the community pharmacists in the present study is shown in Table 2.

**Table 2:** Community pharmacists who fulfilled the practice principles of pharmaceutical care (N = 100)

Components of each pharmaceutical practice principle	Frequency (%)	Frequency (%) of pharmacist who practised all the components
**Patients’ data collection**		0 (0.0)
Who the medicine is for?	38 (38.0)^a^	
Age of patient?	17 (17.0)	
Symptoms of cough (i.e. dry or phlegm cough)?	97 (97.0)	
Any other symptoms?	29 (29.0)	
How long the patient has the cough?	27 (27.0)	
What medication has been taken for the current cough?	10 (10.0)	
Any known allergy to any medications?	8 (8.0)	
Does the patient have other medical conditions/history?	25 (25.0)	
What other medications the patient is taking?	13 (13.0)	
Lifestyles such as occupation, smoking or other daily activities	22 (22.0)	
**Medical information evaluation**		13 (13.0)
The possibility of medication-induced cough was identified	9 (9.0)	
A possible non-medication-induced cough was explained	8 (8.0)	
**Formulating a drug therapy plan**		15 (15.0)
Pharmacist recommended medication(s) for cough	98 (98.0)	
Pharmacist suggested a non-medicine treatment plan	6 (6.0)	
Referred to a doctor due to cough duration or medication-related problem	11 (11.0)	
**Implementing a drug therapy plan by providing counselling on the following** ^b^		0 (0.0)
Possible cause of cough	12 (12.2)	
Name of medication^c^	13 (13.3)	
Dose of the medication^c^	85 (86.7)	
Frequency of medication to be taken^c^	88 (89.8)	
Duration of the therapy	3 (3.1)	
Side effects or precautions^d^	0 (0.0)	
Mechanism of action	3 (3.1)	
Storage conditions of the medicine	0 (0.0)	
Non-pharmacological management	6 (6.1)	
Advice to visit a health care provider if symptoms persist	11 (11.2)	
**Monitoring and modifying the plan**		3 (3.0)
Pharmacists provided contact details and requested the client to return for a follow-up	3 (3.0)	
Pharmacists requested the client’s contact details for follow-up	0 (0.0)	

^a^ Before being prompted whether the medication is suitable for the simulated client’s father

^b^ n = 98 as 2 pharmacists did not recommend any medication or product

^c^ With both label and verbal communication

^d^ Only if a sedative medication was given

Ten questions which should be asked by the community pharmacists were included under patients’ data collection (Table 2). The number of questions asked by the community pharmacists ranged from 0 to 8. As the data were not normally distributed, the median (IQR) of 2.00 (3.00) questions asked was generated. None of the community pharmacists asked the SC all the questions required before deciding on the recommendation.

Of the 100 community pharmacists, three did not ask any questions before recommending medications to the SC while one pharmacist still did not ask any questions even though the SC tried to prompt him/her by asking whether the medication could be used by the SC’s father.

Two of the pharmacists did not recommend any medication or product to the SC as one of them suspected that the cough could be medication-induced and suggested that SC should bring the father’s medications to the pharmacy before he/she could recommend anything. The other pharmacist refused to recommend any medication and advised SC to bring the father to see a medical doctor.

In addition, 62 community pharmacists (62%) assumed that the cough medication was for SC but 22 of these pharmacists realized that the medication requested was for SC’s father upon further questioning while another eight pharmacists only realized this when they were asked by SC whether the medication could be used by his father. However, the remaining 32 pharmacists continued to recommend medication as though SC was the patient even after being prompted if the medication could be used by the SC’s father.

Of the 98 community pharmacists who recommended a medication, 92 (93.9%) initiated counselling on the medication without being asked by the SC, but none of the pharmacists provided all the 10 counselling elements under the practice of implementing the drug therapy plan principle (Table 2). The number of counselling elements provided by these pharmacists ranged from 0 to 6, with a median (IQR) of 2.00 (1.00).

### Number of clients and time spent on consultation

During the SC visits, 74 community pharmacies (74%) did not have any other clients in the pharmacies while 26% had 1 to 9 clients. However, 63% of the community pharmacists spent only one minute or less while the other 37% spent more than one minute but less than four minutes responding to the illness presented by SC (Table 3).

**Table 3:** Time spent on consultation by the pharmacists (N = 100)

Time spent	Percentage of pharmacist
Not more than 1 min	63
Not more than 2 min	29
Not more than 3 min	6
Not more than 4 min	2

The study could not report the exact time spent in consultation with the pharmacists as SC was only using a mobile phone timer and could only turn it off when away from the pharmacists, to avoid arousing any suspicion.

### Medications recommended for cough

A total of 112 medications or products, which consisted of 36 different brands (Additional file 3) were recommended by the community pharmacists and purchased by the SC during the study. Three of the recommended medications did not have any name indicated. Various dosage forms of medications or products were recommended by community pharmacists, with Tussidex Forte™ (dextromethorphan hydrobromide) linctus being the most common.

Medicines containing dextromethorphan were the most commonly recommended for the treatment of dry cough (49%), followed by cloperastine (17.4%), pholcodine (15.3%), and diphenhydramine (6.1%). Among the 98 community pharmacists who recommended a medication(s) or product(s) to the SC, 86 (87.8%) recommended one medication or product, while another 10 (10.2%) recommended two, and two (2%) recommended three medications or products. The median (IQR) cost of treatment was RM10.00 (8.10) and ranged from RM2.00 to RM39.80.

### Appropriateness of medicine recommended

Based on the online Martindale database, cough suppressants such as pholcodine, dextromethorphan and cloperastine hydrochloride, as well as sedative antihistamines such as diphenhydramine in compound preparations, are commonly used and indicated for the complaints mentioned in the simulated case. The appropriateness of the medications recommended was evaluated based on the indications for dry cough [27, 28]. The medication should not impair the ability to drive or operate heavy machinery since the SC’s father is a taxi driver, and safe (without contradictions or precautions) for use in patients with hypertension, hyperlipidaemia and diabetes mellitus based on MIMS Drug Reference [29] or the product inserts.

Of the 98 pharmacists who recommended medications to the SC, only 67 (68.3%) recommended appropriate medications for a patient presenting with comorbidities including hypertension, hyperlipidaemia and diabetes mellitus, as well as meet the requirement for someone who may operate vehicles or machinery. However, three (3.1%) pharmacists dispensed the medications as loose tablets or capsules without the name of the medications on the label and explanation. Among those who had recommended appropriate medications, five (7.5%) also recommended other medications or products for indications such as nasal congestion, running nose or phlegm which were not mentioned by the SC and hence, not indicated in this case.

Eleven (11.2%) pharmacists who recommended medications for the cough also advise the father to come and see the pharmacist or to consult a medical doctor if the cough persisted for a period of time. In addition, another two pharmacists who did not recommend any medications but ask SC to bring the father’s medications to the pharmacy or to consult a doctor.

### Factors associated with the number of questions asked and counselling elements provision

Possible characteristics of pharmacists (sex, age group) and pharmacies (type of ownership, location) which might be associated with the number of questions asked were analysed using univariate and multivariate Poisson regression (Table 4). Only the location of the community pharmacy was significantly associated with the number of questions asked by the pharmacists.

**Table 4:** Possible factors associated with the number of questions asked by the pharmacists (n = 98)^a^

	Univariate Poisson regression		Multivariate Poisson regression	
Factors	IRR (95% CI)	p-value	IRR (95% CI)	p-value
**Sex**				
Female	1.188 (0.929, 1.519)	0.170*	1.024 (0.782, 1.339)	0.865
Male	As reference		As reference	
**Age**				
40 and above	1.026 (0.806, 1.304)	0.837	-	-
Below 40	As reference		-	
**Ownership**				
Company/corporate	1.347 (0.987, 1.839)	0.164*	1.375 (0.999, 1.894)	0.148
Individual pharmacist	1.198 (0.907, 1.582)		1.170 (0.878, 1.558)	
Group of pharmacists	As reference		As reference	
**Location**				
Shah Alam	1.607 (0.867, 2.980)	0.023*	1.682 (0.903, 3.135)	0.044**
Subang Jaya	1.374 (0.759, 2.485)		1.470 (0.809, 2.672)	
Klang	1.071 (0.554, 2.071)		1.144 (0.580, 2.254)	
Selayang	1.020 (0.515, 2.020)		1.109 (0.556, 2.209)	
Petaling Jaya	0.923 (0.491, 1.734)		0.981 (0.520, 1.852)	
Kuala Lumpur	0.923 (0.526, 1.618)		0.949 (0.538, 1.676)	
Kajang	0.635 (0.310, 1.301)		0.707 (0.342, 1.462)	
Ampang Jaya	As reference		As reference	
**Number of branches**	1.000 (1.000, 1.001)	0.268	-	-

^a^ Two respondents from other ownership groups of non-pharmacist were excluded from the analysis due to insufficient number for statistical comparison

* p equal or < 0.25

** p < 0.05

IRR - Incidence rate ratio

CI - Confidence interval

The incidence rate ratio (IRR) of the number of questions asked by community pharmacists practising in Shah Alam was 2.378, 1.772 and 1.714 times significantly higher than those in Kajang, Kuala Lumpur and Petaling Jaya, respectively. Whereas, such IRR for those practising in Subang Jaya was 2.078 and 1.548 times significantly higher than those in Kajang and Kuala Lumpur (Table 5).

**Table 5:** Significant associations of pharmacy location and number of questions asked by the pharmacists (n = 98)^a^

Location	IRR (95% CI)	p-value
Shah Alam	2.378 (1.303, 4.341)	0.005**
Subang Jaya	2.078 (1.176, 3.672)	0.012*
Kajang	As reference	-
Shah Alam	1.772 (1.174, 2.674)	0.006**
Subang Jaya	1.548 (1.075, 2.229)	0.019*
Kuala Lumpur	As reference	-
Shah Alam	1.714 (1.052, 2.795)	0.031*
Petaling Jaya	As reference	-

^a^ Two respondents from other ownership groups of non-pharmacist were excluded from the analysis due to insufficient number for statistical comparison

* p < 0.05

** p < 0.01

IRR - Incidence rate ratio

CI - Confidence interval

Possible associations between the characteristics of pharmacists or pharmacies and the number of counselling elements provided are shown in Table 6. However, there were no significant associations.

**Table 6:** Factors associated with the number of counselling elements provided by the pharmacists (n = 96)^a^

	Univariate Poisson regression	
Factors	Odds Ratio (95% CI)	p-value
**Sex**		
Female	1.178 (0.888, 1.562)	0.255
Male	As reference	
**Age**		
Below 40	1.030 (0.777, 1.366)	0.837
40 and above	As reference	
**Ownership**		
Company/corporate	1.209 (0.862, 1.695)	0.544
Group of pharmacists	1.094 (0.799, 1.498)	
Individual pharmacist	As reference	
**Location**		
Shah Alam	2.167 (0.892, 5.264)	0.457
Subang Jaya	1.590 (0.663, 3.810)	
Klang	1.167 (0.448, 3.036)	
Selayang	1.524 (0.596, 3.894)	
Petaling Jaya	1.333 (0.545, 3.262)	
Kuala Lumpur	1.524 (0.665, 3.493)	
Kajang	1.185 (0.464, 3.029)	
Ampang Jaya	As reference	
**Number of branches**	1.000 (0.999, 1.001)	0.434

^a^ Two pharmacists did not recommend treatment and thus their counselling was not studied, 2 respondents from other ownership groups of non-pharmacist owned were excluded from the analysis due to insufficient number for statistical comparison

## Discussion

Based on the practice principles of APhA, the present study found that none of the 98 community pharmacists who recommended treatment were providing adequate pharmaceutical care services to patients seeking self-medication advice and treatment for a cough.

The results of the present study showed that community pharmacists gathered insufficient patient information which included the identity and medical condition of the patient before initiating the drug therapy plan. Three community pharmacists (3%) did not ask any questions but recommended the medications. Nevertheless, the number is lower than the 15 pharmacists (15%) in a similar previous SCM study in the Klang Valley [15]. The difference may be attributed to the different case scenarios used. In the present study, the SC presented a case with a cough in which most pharmacists knew that they have to differentiate between a dry and wet cough (with phlegm) as this will determine the type of cough medication required. Therefore, more pharmacists in the present study would ask about the symptoms (97%) before they recommended a treatment, compared to the previous study which used back pain as the simulated case. Other studies which used simulated clients or patients to request medications from community pharmacists in Australia [30], Canada [31] and Brazil [20] also reported that community pharmacists did not gather adequate patient medical information, leading to inappropriate medication being recommended and a lack of referrals to other health care providers when required.

The present study revealed that only 38% of the community pharmacists asked who the medication was for. This is important as the person requesting the medication may not be the patient. In the present study, the actual patient was the father who would be much older and may have had other medical conditions or been on other medications which necessitate different management. In addition, very few community pharmacists asked for the age of the patient, social histories such as smoking or occupation, other medical conditions, medication history, and other symptoms or allergies to medication. This information is important for the pharmacists to decide on an appropriate drug therapy plan for the patient. Some medications may cause problems in people with medical conditions such as diabetes and hypertension. Medications with sedative effects should be avoided in people whose occupations require them to be alert, including taxi drivers. A majority of the community pharmacists did not conduct adequate patients’ data collection and hence, did not identify, and rectify the existing and prevent potential medication-related problems. In addition, the community pharmacists at certain locations were significantly more likely to ask more questions in gathering patients’ medical information than others. This may be attributed to the different education or training backgrounds of the pharmacists which could not be determined in the present study.

The appropriateness of medications recommended by the pharmacist for the patient’s complaint was determined based on the patient’s medical history and lifestyle in the simulated scenario. Nevertheless, the study showed that 28 community pharmacists (28.6%) recommended inappropriate medications for SC’s father who had hypertension, hyperlipidaemia and diabetes mellitus, and also worked as a taxi driver which could lead to safety concerns for the patient. In addition, five of the community pharmacists who recommended appropriate medications for the patient also recommended medications for symptoms not experienced by the patient. Such unnecessary use of drugs will increase the risk of side effects besides wasting resources [32].

In the present study, the community pharmacists should have referred the patient to a doctor based on the duration of the cough and possible ACEI-induced cough. A checklist should be developed to guide all community pharmacists in responding to symptoms appropriately. This could help to ensure adequate patient data gathering and medical information evaluation so that medication-related problems could be identified before formulating the drug therapy plan. Non-pharmacological management and referral to other appropriate healthcare providers should be considered. Community pharmacists should also play a more active role as health educators by explaining the patients’ health conditions, the possible causes and their management, and eventually empowering the patients to manage their health conditions [10].

For the practice of implementing a drug therapy plan, the community pharmacists did not provide adequate medication counselling to ensure that SC understood how to use the medications recommended. This is similar to another study in Malaysia [17]. Although most of the pharmacists (92%) initiated counselling without being prompted by SC similar to a study in Brazil [20], none of the pharmacists in the present study provided all the stipulated counselling points.

Most community pharmacists (63%) spent only one minute or less on medication counselling, and none spent more than four minutes. This is similar to many other studies where a majority of the community pharmacists spent only 1 to 5 min counselling the patients [33, 34]. Chua et al. [15] also found that community pharmacists spent an average of only one minute with the SC even though no other customers were waiting to see them. A possible explanation is that community pharmacists thought that cough is a common minor health problem which is self-limiting and can be relieved with simple treatment hence, does not require counselling. However, inadequate patient’s data collection procedures or counselling may lead to potential medication-related problems.

The present study concurred with the study by Chua et al. [15] that community pharmacists should make more effort in medication counselling and not just on the choice of medications. Even if the patients were prescribed or dispensed the appropriate medications, without sufficient information about the medication, the therapeutic objectives may not be met [12] and may even lead to severe or fatal consequences. Medications which cause drowsiness may endanger the patient who is a taxi driver. In addition, preferably a cough mixture which does not increase blood glucose levels and will not affect the patient’s blood pressure should be recommended. Monitoring of these health problems should be emphasized. The pharmacists should also advise SC about coming back to the pharmacy or to consult a medical doctor if the cough persisted.

Only three pharmacists in the present study provided their contact details for SC to follow up, but none requested the contact details of SC for a more proactive follow-up. This finding is similar to studies in Jordan [35] and Saudi Arabia [36] where a majority of the community pharmacists did not practise monitoring the drug therapy plan and follow-up of the patients. Perhaps in the present study, most of the community pharmacists did not indicate any follow-up action since the SC’s request was for a cough which was considered a minor health problem and not a chronic disease. However, proper monitoring and follow-up mechanism is the most important component of pharmaceutical care to detect and rectify any undesirable outcomes [1] as well as to ensure that the therapeutic objectives are achieved.

### Limitations of the study

The present study has a few limitations. The first is the simulated client scenario used. The possibility of inadequate pharmaceutical care services provided may be attributed to the presentation of a dry cough, which is usually considered a minor ailment instead of chronic diseases such as diabetes. The community pharmacists may not have conducted a comprehensive gathering of the patient’s information or provided essential counselling elements due to the chosen case study which is a cough. Therefore, the results of the present study may not be extrapolated to chronic or more serious health complaints. The simulated client scenario was designed to observe the practice of pharmaceutical care which included medication counselling by community pharmacists. However, in the present study, two community pharmacists did not recommend any medications and hence, their practice of pharmaceutical care could not be evaluated. In addition, if the community pharmacists had conducted a more detailed patients’ data collection, they would have referred the patient to a doctor and the study would not be able to proceed beyond the third principle of pharmaceutical care practices.

Some community pharmacists may have been suspicious of SC’s intention and hence, Hawthorne effects may have occurred. Nevertheless, such possibility has been minimised with some role-play training and the pilot study on 10 community pharmacies which allowed SC to gain some experience and confidence as well as be more familiar with the study scenario to enable SC to act more naturally. Recall bias by the SC could not be ruled out by having single SC and without utilizing an audio recording device, although SC tried to memorise and record all the information immediately after leaving the community pharmacies.

Lastly, there is a lack of study on pharmaceutical care services provided by community pharmacists in Malaysia based on the five practice principles for pharmaceutical care as stipulated by APhA [2], and categorization of the pharmacists’ response into each specific principle may require further validation and improvement.

## Conclusions

The present study found that community pharmacists in Malaysia were not providing adequate pharmaceutical care services to clients seeking self-medication for a cough. Inadequate provision of pharmaceutical care may cause harm to the patient due to inappropriate recommendations of medications, exposing the patient to existing or potential medication-related problems. The Malaysian Ministry of Health and professional bodies such as the Malaysian Pharmacists Society and the Malaysian Community Pharmacy Guild should develop guidelines for the provision of pharmaceutical care by community pharmacists, to ensure better health care services.

## Electronic supplementary material

Below is the link to the electronic supplementary material.

Supplementary Material 1

Supplementary Material 2

Supplementary Material 3

## Data Availability

The datasets used and/or analysed for the present study are available from the corresponding author upon reasonable request.

## Abbreviations

APhA: American Pharmacists Association
SCM: Simulated client method
SC: Simulated client
ACEI: Angiotensin-converting enzyme inhibitor
OBRA: Omnibus Budget Reconciliation Act
SPSS: Statistical package for the social science
SD: Standard deviation
IQR: Interquartile range
TM: Trademark
MIMS: Monthly index of medical specialties
IRR: Incidence rate ratio
CI: Confidence interval
n.d.: No date
