# Supplementary material for: Practice of pharmaceutical care by community pharmacists in response to self-medication request for a cough: a simulated client study
Source: BMC Health Serv Res. 2023 Jun 20;23:657. doi: 10.1186/s12913-023-09642-x (PMC10283233; doi:10.1186/s12913-023-09642-x)
Supplement: Supplementary file 1 — Supplementary Material 1 [file 12913_2023_9642_MOESM1_ESM.docx]

**Additional File 1**

**Guide with standard answers for SC when asked by pharmacists**

| **Questions asked by pharmacists** | **Standard answers from SC** |
| --- | --- |
| 1. Type of cough? | Dry cough. |
| 2. Who was the medicine for? | Father. |
| 3. What was the father's age? | 60. |
| 4. Duration of the cough? | On and off for the last 2 months. |
| 5. Any other symptom like running nose, sore throat, fever or pain? | None. |
| 6. Was the father smoking? | Father was a smoker. |
| 7. What was the father's occupation? | A taxi driver. |
| 8. What action or medicine has been taken? | Only took Breacol^TM^ to relieve his cough, but the cough has persisted. |
| 9. Medical history? | Hypertension, hyperlipidaemia and diabetes mellitus diagnosed 2 years ago by a doctor in a government hospital. |
| 10. Current medications? | Unsure the name of the medications and when they were initiated. Only know that they were for high blood pressure, diabetes and high cholesterol and were being taken daily by the father. |

Breacol^TM^ : contains guanfenesin^a^

^a^MIMS Malaysia. MIMS Drug Reference 157th Ed. Wan Chai, Hong Kong: MIMS (Hong Kong) Limited. 2019.

SC - Simulated client

TM - Trademark
