# Supplementary material for: Practice of pharmaceutical care by community pharmacists in response to self-medication request for a cough: a simulated client study
Source: BMC Health Serv Res. 2023 Jun 20;23:657. doi: 10.1186/s12913-023-09642-x (PMC10283233; doi:10.1186/s12913-023-09642-x)
Supplement: Supplementary file 2 — Supplementary Material 2 [file 12913_2023_9642_MOESM2_ESM.docx]

**Addition File 2**

**Fulfilment of pharmaceutical care practice principles**

| **Pharmaceutical care practice principles** | **Component number** | **25 components of pharmaceutical care**  **practice** | **Positive response required** |
| --- | --- | --- | --- |
| **1. Patients' data collection** | 1 | Who the medicine is for? | All |
|  | 2 | Age of patient? |  |
|  | 3 | Symptoms of cough (i.e. dry or phlegm cough)? |  |
|  | 4 | Any other symptoms? |  |
|  | 5 | How long the patient has the cough? |  |
|  | 6 | What medication has been taken for current cough? |  |
|  | 7 | Any known allergy to any medications? |  |
|  | 8 | Does the patient have other medical conditions/history? |  |
|  | 9 | What other medications the patient is taking? |  |
|  | 10 | Other information, i.e. smoking, occupation, medicine specifications. |  |
| **2. Medical information evaluation** | 11 | Identified the possibility of medication-induced cough | Either one |
|  | 12 | Explained possible cause of cough (other than medication-induced) |  |
| **3. Formulating a drug therapy plan** | 13 | Pharmacist recommended medication(s) for cough | Either 13 and 14 or 15 |
|  | 14 | Pharmacist suggested a non-medicine treatment plan (i.e. changes of lifestyle or diet) |  |
|  | 15 | Pharmacist chose to refer to the doctor due to the duration of cough or medication-related problem |  |
| **4. Implementing a drug therapy plan** | 16 | Name of medication | All |
|  | 17 | Dose of the medication |  |
|  | 18 | Frequency of medication to be taken |  |
|  | 19 | Duration of the therapy |  |
|  | 20 | Side effects or precautions |  |
|  | 21 | Mode of action of medication |  |
|  | 22 | Storage |  |
|  | 23 | To see doctor if the cough persists for more than a certain time period or after finishing the medications given |  |
| **5. Monitoring and modifying the plan** | 24 | Request client to come back for the follow-up | Either one |
|  | 25 | Request client’s contact for the follow-up |  |
