# Supplementary material for: Practice of pharmaceutical care by community pharmacists in response to self-medication request for a cough: a simulated client study
Source: BMC Health Serv Res. 2023 Jun 20;23:657. doi: 10.1186/s12913-023-09642-x (PMC10283233; doi:10.1186/s12913-023-09642-x)
Supplement: Supplementary file 3 — Supplementary Material 3 [file 12913_2023_9642_MOESM3_ESM.docx]

**Additional File 3**

**List of medicines or products recommended by community pharmacists**

| **Brand name** | **Dosage form** | **Medicine or product ingredients** | **Number of pharmacists** |
| --- | --- | --- | --- |
| Altra | Tablet | Montelukast | 1 |
| Aspira | Tablet | Montelukast | 1 |
| Avaphen | Syrup | Diphenhydramine HCl + Ammonium Chloride | 1 |
| Axcel Dextromethorphan | Syrup | Dextromethorphan Hydrobromide | 2 |
| B-ligno | Capsule | Cordyceps Sinensis + Lignosus Rhinocerus | 1 |
| Bena Expectorant | Syrup | Diphenhydramine HCl + Ammonium Chloride | 2 |
| Bencodyl Linctus | Linctus | Diphenhydramine HCl + Ammonium Chloride | 1 |
| Bromelain enzyme | Powder | Bromelain | 1 |
| Cetirizine STADA | Tablet | Cetrizine | 1 |
| Cofcare | Syrup | Adhatoda vasica + Other Herbs | 1 |
| Copastin | tablet | Cloperastine Hydrochloride | 17 |
| Cough-en Rx | Linctus | Dextromethorphan Hydrobromide + Phenylephrine Hydrochloride + Triprolidine Hydrochloride | 1 |
| Dexcophan Plus | Linctus | Dextromethorphan Hydrobromide + Ephedrine Hydrochloride + Ammonium Chloride | 2 |
| Dextrophan | Syrup | Dextromethorphan Hydrobromide | 2 |
| Ducodin Forte | Linctus | Pholcodine | 1 |
| Duro-Tuss Expectorant | Liquid | Pholcodine + Bromhexine HCl | 2 |
| Duro-Tuss Forte | Liquid | Pholcodine | 10 |
| Duro-Tuss Regular | LIquid | Pholcodine | 1 |
| Echinacea | Tablet | Echinacea | 1 |
| Fluimucil A | Effervescent Tablet | N-Acetylcysteine | 1 |
| Hosolvon | Elixir | Bromhexine HCl | 3 |
| Koflet | Syrup | Adhatoda vasica + Other Herbs | 1 |
| Levocetirizine | Tablet | Levocetirizine | 1 |
| Loratadine STADA | Tablet | Loratadine | 2 |
| Pabron cough | Syrup | Carbocisteine | 1 |
| Proezine | Tablet | Promethazine | 2 |
| Prospan | Syrup | Ivy Extracts | 1 |
| Sedilix-Rx Linctus | Linctus | Dextromethorphan Hydrobromide + Phenylephrine Hydrochloride + Promethazine Hydrochloride | 1 |
| Stopcof | Syrup | Adhatoda vasica + Other Herbs | 2 |
| Sunex cough syrup | Syrup | Diphenhydramine HCl + Ammonium Chloride | 1 |
| Sunthorphan | Syrup | Dextromethorphan Hydrobromide | 1 |
| Telfast | Tablet | Fexofenadine HCl | 1 |
| Tussedyl Forte | Syrup | Pholcodine | 1 |
| Tussidex Forte | Linctus | Dextromethorphan Hydrobromide | 39 |
| Uphadyl Forte Expectorant | Syrup | Diphenhydramine HCl + Ammonium Chloride | 1 |
| V-lief | Syrup | Ivy Extracts | 1 |
| Unknown 1^a^ | Tablet | Unidentified | 1 |
| Unknown 2^a^ | Capsule | Unidentified | 2 |

^a^Not labelled or mentioned by the pharmacist

Cl - Chloride; HBr - Hydrobromide; HCl - Hydrochloride
